# Patterns of e-cigarette use and interest in cessation among current users in Ontario: An online cross-sectional study

**DOI:** 10.1371/journal.pone.0322736

**Published:** 2025-05-09

**Authors:** Javad Heshmati, Kerri-Anne Mullen, Evyanne Quirouette, Jordan Bernick, Andrew Pipe, Hassan Mir

**Affiliations:** 1 Division of Cardiology, University of Ottawa Heart Institute, Ottawa, Canada; 2 Ottawa Model for Smoking Cessation, Ottawa, Canada; 3 Faculty of Medicine, University of Ottawa, Ottawa, Canada,; 4 School of Epidemiology and Public Health, University of Ottawa, Ottawa, Canada,; 5 Cardiovascular Research Methods Centre, Department of Medicine, University of Ottawa Heart Institute, Ottawa, Ontario, Canada; National Institutes of Health, University of the Philippines Manila/De La Salle University, PHILIPPINES

## Abstract

Use of electronic cigarettes, commonly referred to as vaping, has increased substantially in recent years. Our present study aimed to explore vaping patterns and factors that may associated with intention to quit among participants in Ontario, Canada. In this original survey, a total of 757 participants over the age of 15 and currently using e-cigarettes were invited to complete an online survey via social media advertisement about vaping. We included participants who used any type of vaping device. Binary logistic regression modelling was applied to assess patterns of vaping with intention to quit. Of the 757 participants, 44.2% were under 25 years old, and 57.2% were male. Notably, 29.1% of respondents tried vaping before 18 years of age and 34.6% vaped regularly at 18 or younger. Of the sample, 81.4% reported vaping daily and 63.1% vaped more than 10 times a day. Almost half (49.6%) reported consuming more than 10 mg of nicotine per day, 23.8% were consuming more than 20mg of nicotine per day, and 6.2% were consuming more than 40mg of nicotine per day. Among respondents, 41.3% were interested in quitting. Regression analysis revealed that younger individuals, those consuming nicotine, and those with higher levels of nicotine dependence were more inclined to quit. Additionally, those who perceive vaping to be more harmful and more addictive showed a greater intention to quit. Conversely, individuals who perceive vaping to be less harmful or addictive than smoking were significantly less likely to quit. Our results also indicated that those who were vaping to quit smoking (42.7%), vaping socially (40.4%) and vaping to manage a mental health issue (37.2%) had the highest intention to quit. Those who vaped as it was healthier than smoking (32.3%), due to the flavours (33.6%), and financial reasons (34.5%) were least likely to want to quit. This study confirms that a large portion of e-cigarette users are young, daily users with high nicotine use and dependence. Given the high interest to quit vaping, there is a need for continued education, health promotion, prevention, and targeted treatment programs.

## 1 Introduction

E-cigarettes are a battery-operated device that heats a liquid solution to produce an inhalable aerosol. This liquid and associated aerosol may or may not contain nicotine [1,2]. While these devices can be used by adult smokers to help them quit smoking tobacco, recreational use especially among youth and young adults, has been rising at an alarming rate and serves as a major public health concern [3]. In 2019, the US Centers for Disease Control and Prevention warned users, particularly targeting youth, young adults, and pregnant women [4].

There is growing evidence that e-cigarettes, especially containing nicotine, can serve as a smoking cessation aid [5]. A Cochrane review that is regularly updated reported high-certainty evidence that e-cigarettes with nicotine increase quit rates compared to nicotine replacement therapy. However, this finding is based on seven clinical studies involving 2,544 participants with heterogeneous control groups [6]. Also, results from clinical trials are not population level studies and thus may not be generalizable to those vaping recreationally. While there was no significant increase in adverse events, the longest follow-up was only two years, and few studies achieved this duration. Given this, there remains a lack of consensus on using e-cigarettes as smoking cessation aids.

E-cigarette use and vaping consumption increased from 8.4% to 14.6% within one year, from 2017 to 2018 in Canada [7]. Routine evaluations are vital to understand the impact of e-cigarette policies on use [8]. Before May 2018, the sale and marketing of nicotine-containing e-cigarettes required approval, yet they were widely accessible despite lacking this [9]. With the enactment of the Tobacco and Vaping Products Act (TVPA) in May 2018, nicotine-containing e-cigarettes became legally available for sale, accompanied by expanded advertising and the presence of international brands [10]. This shift increased retail accessibility. Studies have indicated a rise in youth vaping subsequent to the implementation of the TVPA [11].

Evidence-based vaping cessation programs will be crucial for supporting people who vape and are interested in quitting. However, such programs are not widely accepted or evaluated, and existing interventions have not been investigated on a large scale. When designing such programs, it is necessary to gain a better understanding of the demographics, patterns, and willingness to quit among those who are currently vaping, as this can help establish guidelines for treatment [12]. Studies investigating both adolescent and adult e-cigarette users’ intentions to quit reveal a significant desire for cessation [13]. Data from the Wave 4 PATH Study and the 2021 NYTS indicate that a majority of users express intentions to quit e-cigarettes, with approximately 60.7% of adults and nearly two-thirds of adolescents contemplating cessation [14,15].

Understanding factors influencing cessation attempts, such as smoking history and nicotine dependence, is crucial, especially considering the multiple attempts often needed for successful cessation [16,17]. Previous interventions and programs have aimed to educate the target population about vaping risks, however, correlations between these factors and actual cessation behaviors remain uncertain [18,19]. Additionally, given the uncertain long-term consequences of vaping, assessing users’ willingness to break nicotine addiction is paramount for effective cessation interventions [20]. Therefore, the aims of this survey were to investigate patterns of e-cigarette use and factors that may affect willingness to quit among current e-cigarette users.

## 2 Methods

### 2.1 Design

This online cross-sectional survey of current e-cigarette users in Ontario was designed by a team led by Dr. Mir at the University of Ottawa Heart Institute’s Ottawa Model for Smoking Cessation Program. The survey incorporated patient demographics, patterns of e-cigarette, knowledge and perception of e-cigarettes, co-use of other addictive substances, level of addiction as measured by a modified Fagerstrom score for nicotine dependence, and interest in quitting. A copy of the survey and invitation to participate in the survey posters on Facebook is included in the S1 File. This study was approved by the Ottawa Health Science Network Research Ethics Board (20210588-01H).

### 2.2 Recruitment

The recruitment period started on January 17, 2022, and ended on February 28, 2022. Individuals over the age of 15, currently using e-cigarettes at least once a week, and able to speak English or French were invited to complete the voluntary and anonymous survey. We used convenience sampling via social media to enrich the study population of those who use e-cigarettes. The survey was shared using sponsored advertisements composed of varying imagery, messages, and framing on Facebook, Instagram, and Tik Tok. Participants were recruited from Ontario, Canada.

### 2.3 Inclusion/exclusion criteria

Inclusion criteria for this survey were (1) Age: ≥ 15 years old; (2) Current regular e-cigarette user, defined as those who vaped at least once a week for the past four weeks; (3) Ability to read in English or French. Those not meeting inclusion criteria were excluded.

### 2.4 Procedure

A survey was administered using sponsored advertisements on Facebook, Instagram, and TikTok. Through this, the social media platforms delivered advertisements to the screens of targeted audiences using proprietary marketing algorithms that utilized users’ self-reported interests. The user could click and engage with the advertisement, which led them to a landing page that provided additional information about the study and a link to the survey. A convenience sampling method was also used by promoting the survey through Tweets and Facebook posts, which briefly noted the inclusion criteria, purpose of the survey, and encouraged participation in the survey and sharing of the social media posts to maximize reach. This strategy aimed to enrich the study population with regular e-cigarette users to understand their knowledge, perceptions, and patterns of use.

The initial screening questions and the informed consent page appeared before the survey questions were shown within REDCap. The screening questions asked whether participants 1) had vaped at least once a week for the past four weeks 2) were 15 years of age or older and 3) lived in the Ontario region. If the participant clicked yes answer to all three questions was yes, then they were shown the informed implied consent page, which explained the purpose of the survey and included language stating that IP addresses and other identifying information would not be collected or linked to them. Respondents who provided implied consent by clicking “continue” at the bottom of the consent page were redirected to the survey hosted through the REDCap online survey platform. REDCap is a secure web application for building and managing online surveys and databases [21]. Survey items assessed vaping history and patterns of use (e.g. age of initiation, frequency of use), reasons for vaping, intention to quit, perceptions of health risks associated with vaping, and use of other substances”. Those below the age of 15 were not included in this study.

Individuals who completed the survey were offered a $10 gift card to Tim Hortons. To confirm their identity, a unique automated code was generated by REDCap and provided to the participant upon completion of the survey. This was emailed to the study team, and once confirmed, a gift card (virtual or physical) was sent to their preferred address.

### 2.5 Statistical analysis

The collected data were analyzed using SPSS 22.0 software (IBM, NY, USA). Descriptive statistics were computed using percentages and means. Logistic regression was utilized to model and predict vaping intention. Two-tailed probability values of 0.05 or less were considered statistically significant. We assessed the notable correlation between variables and the intention to quit as our primary variable of interest. Variables that show a significant correlation with the intention to quit outcome and have a causal relationship with this outcome are considered confounding variables. Odds ratios (ORs) greater than 1 indicate a heightened intention to quit, while ORs less than 1 indicate a diminished intention to quit. We have adjusted for age, sex, first-time vaping ever, first time of regular vaping, nicotine content of the vaping device, tobacco smoking, cannabis consumption, and Fagerstrom score in a multivariable logistic regression model.

The Fagerstrom Test for Nicotine Dependence (FTND) is a widely used instrument to measure the intensity of physical addiction to nicotine [22]. It helps in assessing the level of dependence on nicotine in individuals who smoke cigarettes [23]. Research has demonstrated that the Fagerstrom Test for Nicotine Dependence (FTND) can be effectively employed and validated for assessing dependence on vaping [24,25]. The Fagerstrom Test consists of six questions that ask about the frequency and intensity of smoking (vaping), the urge to vape, and dependence symptoms. Each question has multiple-choice answers, and based on the responses, a score is calculated. The highest attainable score is 10 points, with higher scores indicating a greater dependence on nicotine. Total amount of nicotine consumed per day was calculated by multiplying the amount of nicotine consumed (300 puffs are approximated to be 1ml) by the average concentration of the nicotine consumed by the user.

## 3 Results

### 3.1 Demographics

All participants were recruited via social media with sponsored advertisements for those living in Ontario. A total of 757 participants completed the survey over a 6-week period between January 17, 2022 and February 28, 2022. A total of $4,209.59 was spent on paid advertisement over the 6-week sampling period. This resulted in 90,507 impressions (number of times one of our ads was seen by a user). Given we had 757 completed surveys, 0.84% of individuals who saw our ad completed our survey. Of this sample, 44.2% were younger than 25 years old, while 55.8% were 25 or older. Among these respondents, 57.2% were male and 39.9% were female and 2.9% prefer not to answer this question. Additionally, 2.9% of individuals did not prefer to disclose their sex and 4.8% preferred not to disclose their gender. The demographic characteristics of participants are reported in **Table 1**.

**Table 1:** Demographics and intention to quit by category.

	N(%)	Intention to quit (%)*	
		Yes	No
Total	757(100.0)	313(41.3)	444(58.7)
Age (Years)			
15 - 17	76(10.0)	24(31.6)	52(68.4)
18 - 24	259(34.2)	125(48.3)	134(51.7)
25 - 34	170(22.5)	69(40.6)	101(59.4)
35 - 44	133(17.6)	61(45.9)	72(52.4)
45 - 54	58(7.7)	20(34.5)	38(65.5)
55 - 64	48(6.3)	13(27.1)	35(72.9)
65+	13(1.7)	1(7.7)	12(92.3)
Biological Sex			
Male	433(57.2)	188(43.4)	245(56.6)
Female	302(39.9)	117(38.7)	185(61.3)
Prefer not to disclose	22(2.9)	10(45.5)	12(54.5)
Gender			
Male	435(57.5)	188(43.2)	247(56.8)
Female	286(37.8)	112(39.2)	174(60.8)
Prefer not to disclose	36(4.8)	13(36.1)	23(63.9)

*Intention to quit percent is calculated by row.

### 3.2 Pattern *of* vaping

Notably, 29.1% of respondents tried vaping before 18 years of age and 34.6% vaped regularly at 18 or younger. Almost two-thirds of participants (65.3%) vaped for the first time before the age of 30 and 74.5% began vaping regularly by the age of 35 (S1 File). Younger individuals were more likely to want to quit than older individuals. Of the sample, 81.4% reported vaping daily and 63.1% vaped more than 10 times a day. Only 16.2% vaped for less than a year while 71.0% had vaped for 1 to 5 years and 12.8% had vaped for over 5 years. Those who had vaped for fewer years (less than 5 years) were more likely to want to quit.

When comparing frequency of vaping, 37.0% reported fewer than 10 sessions of vaping per day (10 minutes or 10–15 puffs is equal to 1 session; this is the approximate amount of nicotine obtained from 1 cigarette but depends on the e-cigarette nicotine concentration) [26,27]. An additional 31.6% reported 10 to 19 sessions per day and 31.5% reported 20 or more sessions per day. Those vaping more often were more interested in quitting. Nearly half (49.6%) reported more than 10 mg of nicotine per day, 23.8% were using more than 20mg of nicotine per day and 6.2% were using more than 40mg of nicotine per day. Of note, 14.4% of respondents reported consuming 0 mg of nicotine per day or they were not sure about the amount of nicotine in their devices. Those consuming higher amounts of nicotine were more likely to want to quit compared to those that did not consume nicotine in their e-cigarette. The Fagerstrom nicotine dependence score was calculated based on responses; 23.4% had a very low (score of 0–2), 25.8% had a low (score of 3–4), 15.6% had a moderate (score of 5–6), 21.8% had a high (score of 7–8), and 13.5% had a high (score of 9–10) level of nicotine dependence. Those with high levels of dependence were more interested in quitting e-cigarettes.

### 3.3 Perceptions *of* vaping

**Fig 1:**
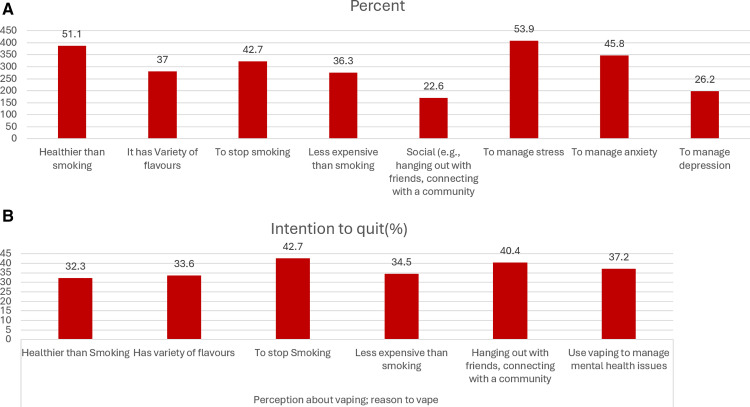
Reasons for vaping among participants (A) and intention to quit based on reasons for vaping (B).

Respondents reported several reasons for vaping, which included quitting smoking cigarettes (42.7%). Other factors included vaping being heathier (51.1%), having more flavours (37.0%), and being less expensive (36.3%) than smoking. A smaller portion (22.6%) reported vaping due to social reasons. Mental health was the most cited reason for vaping. Over 60.0% of individuals reported that they used e-cigarettes to manage a mental health issue (53.9% to manage stress, 45.8% to manage anxiety, and 26.2% to manage depression (26.2%). See **Fig 1A** for details. Those who were vaping to quit smoking (42.7%), vaping socially (40.4%) and vaping to manage a mental health issue (37.2%) had the highest intention to quit. Those who vaped as it was healthier than smoking (32.3%), due to the flavours (33.6%), and financial reasons (34.5%) were least likely to want to quit (Fig 1B**).**

**Fig 2:**
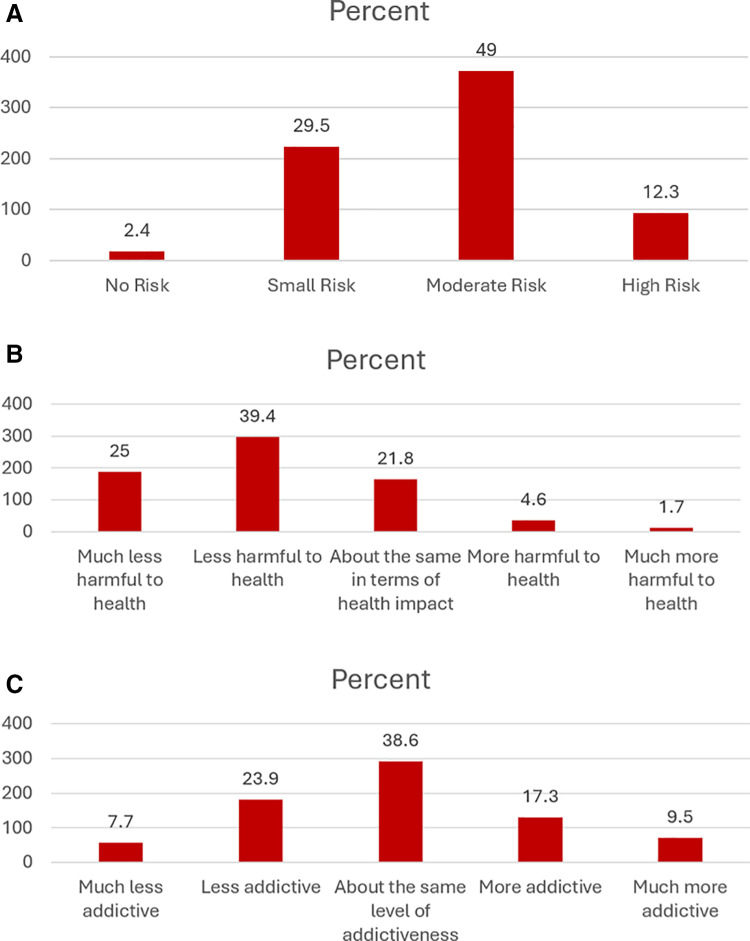
Perception of vaping among participants; Health risk from vaping (A), harms from vaping compared to smoking (B), and addictiveness of vaping compared to smoking (C).

**Fig 3:**
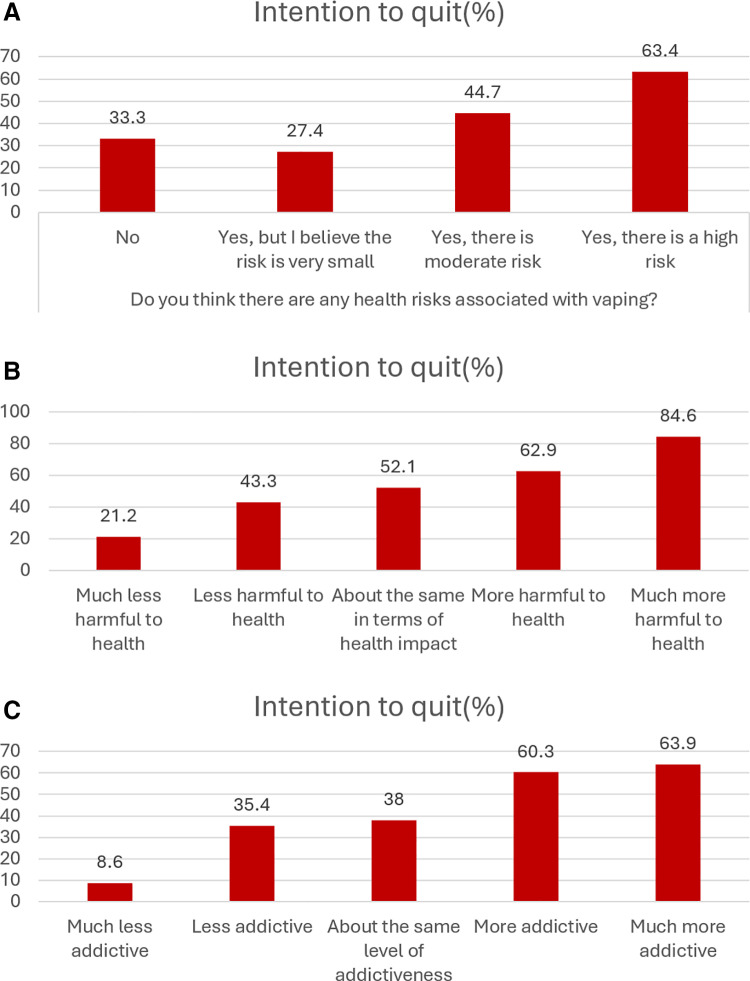
Intention to quit based on perceptions of vaping; Health risk from vaping (A), harms from vaping compared to smoking (B), and addictiveness of vaping compared to smoking (C).

Most respondents appreciate health risks due to vaping. Of the sample, 2.4% felt there was no risk to vaping, 29.5% felt there was a small risk, 49.0% reported a moderate risk, and 12.3% felt there was a high risk to health from vaping (**Fig 2A**). Intention to quit increased as perception of harm increased: 33.3% of those reporting no risk, 27.4% of those reporting small risk, 44.7% of those reporting moderate risk, and 63.4% of those reporting high risk (Fig 3A**).**

Most respondents feel that vaping is less harmful that smoking. When comparing the harms of vaping to smoking, 64.4% reported that it was less harmful to health; 28.1% reported it was the same or more harmful to health (**Fig 2B**). Intention to quit increased as perception of harm of vaping compared to smoking increased: 21.2% of those reporting much less harm, 43.4% of those reporting less harm, 52.1% of those reporting the same harm, 62.9% of those reporting more harm, and 84.6% of those reporting much more harm (**Fig 3B**).

More respondents felt that vaping is less addictive than smoking: 31.6% felt vaping was less addictive than smoking, 38.6% felt it was as addictive, and 26.8% felt it was more addictive (**Fig 2C**). Intention to quit increased as perception of addictiveness from vaping compared to smoking increased: 8.6% of those reporting much less addictiveness, 35.4% of those reporting less addictiveness, 38.0% of those reporting the same addictiveness, 60.3% of those reporting more addictiveness, 63.9% of those reporting much more addictiveness (**Fig 3C).**

### 3.4 Co-use *of* substances

Tobacco use was reported by 48.2% of respondents. Of those that smoke, approximately 25.0% were daily smokers (11.9% of total sample) and 53.0% smoked at least once a week (25.5% of total sample). Alcohol is the most co-used substance with almost 70.0% of individuals consuming at least 1 drink a week. Of those that drink, 33.0% consume more than 7 drinks a week (23.1% of total sample) and 18.0% consume 13 or more drinks per week (12.5% of total sample). Cannabis is also commonly consumed with over two-thirds of participants (67.2%) reporting ever cannabis use. Of those that use cannabis, half (50.0%) are daily users (33.8% of total sample) and 78.0% use cannabis at least once a week (52.7% of total sample). While less common, 36.2% of participants reported use of other recreational substances such as sedatives, opioids, stimulants, or cocaine in the past 6-months. Co-use of other substances (tobacco, alcohol, cannabis, and recreational drugs) is reported in **Table 2**. Co-use of substances increased intention to quit: 44.2% of those who consume alcohol compared to 35.0% of those who do not; 47.9% of those who smoke tobacco compared to 35.2% of those who do not; 43.2% of those who consume cannabis compared to 37.5% of those who do not. Of those who have co-used any of these substances in the past 6 months, 41.9% were interested in quitting; 35.1% of those who have not co-used any of these substances were interested in quitting. See **Table 2** for additional details.

**Table 2:** Co use of substances (tobacco, alcohol, cannabis, recreational drugs of any sort) and intention to quit by category.

	N(%)	Intention to quit (%)*	
		Yes	No
	Frequency of Alcohol consumption (drinks)		
None	234(30.9)	82(35.0)	152(65.0)
1 - 3	233(30.8)	102(43.8)	131(56.2)
4 - 7	115(15.2)	55(47.8)	60(52.2)
8 - 12	80(10.6)	40(50.0)	40(50.0)
≥13	95(12.5)	34(35.8)	61(64.2)
Frequency of tobacco smoking			
Every day	90(11.9)	47(52.2)	43(47.8)
Every 2–3 days	41(5.4)	20(48.8)	21(51.2)
Once a week	62(8.2)	34(54.8)	28(45.2)
Once a month	50(6.6)	23(46.0)	27(54.0)
Less than once a month	122(16.1)	51(41.8)	71(58.2)
Do not Smoke	392(51.8)	138(35.2)	254(64.8)
Frequency of cannabis consumption			
Every day	256(33.8)	104(40.6)	152(59.4)
Every 2–3 days	106(14.0)	51(48.1)	55(51.9)
Once a week	37(4.9)	14(37.8)	23(62.2)
1 - 3 times a month	40(5.3)	17(42.5)	23(57.5)
Less frequently than once a month	70(9.2)	34(48.6)	36(51.4)
Do not consume cannabis	248(32.8)	93(37.5)	155(62.8)
Use of recreational drugs (past six months)			
Sedatives (e.g., Ambien, Xanax, Lorazepam/Ativan)	70(9.2)	27(38.6)	43(61.4)
Opiates (e.g., Oxycontin, fentanyl, heroin)	41(5.4)	36(63.4)	15(36.6)
Stimulants other than caffeine (e.g., Concerta, Adderall, Speed)	101(13.3)	51(50.5)	50(49.5)
Cocaine	63(8.3)	40(63.5)	23(36.5)
None of the above	533(63.8)	203(38.1)	330(61.9)
Ever used any kind of alcohol, tobacco, cannabis or recreational drugs (past six months)	700(92.5)	293(41.9)	407(58.1)
Never used any kind of alcohol, tobacco, cannabis or recreational drugs (past six months)	57(7.5)	20(35.1)	37(64.9)

*Intention to quit percent is calculated by row.

### 3.5 Correlates *of* having quit vaping

The crude and adjusted logistic regression results are shown in S1 File. The primary outcome of interest was the intention to quit. Adjustment was performed for age, sex, age when they tried vaping, age when they started vaping regularly, nicotine content in their vaping device, frequency of vaping, total nicotine content consumed, perceptions about e-cigarette, tobacco use, cannabis use, and the Fagerstrom nicotine dependence score. When using the 15- to 17-year-old respondents as the reference, younger individuals were significantly more likely to want to quit vaping compared to older individuals in both crude and adjusted analysis (18–24yo adjusted OR 2.7, 95% CI 1.35–5.45, p-value 0.005; 25–34yo adjust OR 3.9, 95% CI 1.45–10.49, p-value 0.007; 34–44yo adjusted OR 5.8, 95% CI 1.64–20.41, p-value 0.006). Those over the age of 55 trended towards being less likely to want to quit but this was not statistically significant (55–64yo adjusted OR 0.5, 95% CI 0.05–4.80, p-value 0.55; 65 + yo adjusted OR 0.09, 95% CI 0.003–2.25, p-value 0.14).

When comparing those without nicotine in their e-cigarettes, those with nicotine were more likely to want to quit vaping on both crude and adjusted analyses. Adjusted analyses were statistically significant for those with 3–15mg/ml (adjusted OR 2.7, 95% CI 1.30–5.59, p-value 0.008) and 16–24mg/ml (adjusted OR 3.1, 95% CI 1.48–6.48, p-value 0.003). Those with higher levels of nicotine in their e-liquid trended towards increased odds to want to quit but this was not statistically significant.

When compared to those who believe vaping is more harmful to health than smoking, those who believe that vaping is less harmful are also less likely to want to quit on crude (OR 0.24, 95% CI 0.13–0.46, p-value <0.001) and adjusted analysis (adjusted OR 0.37, 95% CI 0.17–0.82, p-value 0.013). Those who felt smoking and vaping are similar in their harms were also less likely to intend to quit on crude analysis, but this was not significant when adjusted. When compared to those who believe vaping is more addictive than smoking, those who believe it is similar or less addictive were also less likely to intend to quit (adjusted OR 0.37, 95% CI 0.24–0.58, p-value <0.001 and adjusted OR 0.26, 95% CI 0.16–0.43, p-value <0.001 respectively).

When comparing those at very low nicotine dependence based on the Fagerstrom score, there was a significantly higher likelihood of wanting to quit for those at higher levels of dependence in both crude and adjusted analyses (low level, adjusted OR 1.9, 95% CI 1.11–3.16, p-value 0.02; moderate level, adjusted OR 2.5, 95% CI 1.31–4.69, p-value 0.005; high level, adjusted OR 2.3, 95% CI 1.21–4.27, p-value 0.01). Those at very high level of dependence were significantly more likely to want to quit on crude analysis (crude OR 2.29, 95% CI 1.37–3.81, p-value 0.001) but this was no longer significant when adjusted for other variables in the logistic regression (adjusted OR 2.0, 95% CI 0.90–4.54, p-value 0.086). There was no significant effect based on gender, age when they first tried vaping, age when they started vaping regularly, the number of times the respondent vapes, tobacco use, or cannabis use.

## 4 Discussion

This original online cross-sectional study surveyed current e-cigarette users to better understand their knowledge, perception, patterns of use, and intention to quit. To the best of our knowledge, this is among the first studies in Ontario, Canada, aimed at assessing factors that influence vaping patterns and intention to quit.

There are several key findings from this study. Those using e-cigarettes were younger with almost half being under age 25 and two-thirds being under age 35. It highlights concerns that e-cigarettes are being used recreationally among youth and young adults rather than a smoking cessation aid among older individuals. These results align with previous research [28,29], indicating that nearly 75.0% of participants start vaping regularly before the age of 35. A majority of respondents (87.0%) in the current study started vaping within the past 5 years, aligning with the rising concerns of vaping as a growing public health crisis [30,31]. Recent studies have shown that vaping among young people in Canada has increased by approximately 50.0% [32]. Only 40.0% of individuals reported using e-cigarettes to quit smoking tobacco. This matches recent studies that show 25–40% of individuals use e-cigarettes as a smoking cessation aid while 30–50% of individuals use e-cigarettes recreationally [33,34]. It is important to note that e-cigarettes are not an approved or regulated cessation aid in Canada [35] and their long-term efficacy and safety is unclear [36]. Over 60.0% of individuals are using e-cigarette as a means of managing a mental health issue. This is an area of concern as e-cigarettes are not approved for this indication, use may add nicotine dependence and addiction to their other mental health issues [37], and perceived effectiveness of e-cigarettes to manage mental health may result in individuals being less likely to seek evidence-based mental health care [38,39]. This trend aligns with findings from prior research [40,41], indicating that psychological interventions, counseling, and stress management techniques could play pivotal roles in reducing the prevalence of vaping and deterring individuals from engaging in alternative forms of smoking. This study shows that almost 90.0% of e-cigarette users have devices containing nicotine, many of whom are consuming remarkably high levels of nicotine (almost half using more than 10.0 mg/day, close to one-quarter using more than 20.0 mg/day, and 6.0% using more than 40.0 mg/day). There was also high nicotine dependence based on the modified Fagerstrom score, which showed that more than half of the respondents had moderate or greater level of nicotine dependence. Use of e-cigarette may serve as an additional form of nicotine addiction in some users, which requires systematic, evidence-based support and intervention to manage [42].

Intention to quit was the primary outcome of this study and over 40.0% of respondents reported interest in quitting within the next 6 months. Adjusted regression analysis revealed that younger individuals (less than age 44) were significantly more likely to want to quit vaping. This aligns with previous research indicating that the intention to quit and actual quit attempts are more common among younger vapers compared to older adults [43]. However, it is important to note that our study primarily included younger participants. Therefore, if older age groups underwent more extensive examination, there may be significant associations that were not readily apparent in our analysis. Regression analysis also demonstrated that those using nicotine in their e-cigarette and those with higher levels of nicotine dependence based on the modified Fagerstrom score were more inclined to quit. However, on adjusted analysis, higher levels of nicotine intake and dependence were no longer statistically significant. This seems to align with previous research, which showed that intention to quit is lower among vapers who are more addicted and experience stronger cravings for nicotine [43]. Another study showed that individuals with higher Fagerstrom scores are also less likely to want to quit during the 6-month follow-up period [44]. One study indicated that an increase of one point in the initial Fagerstrom score lowered the chances of smoking abstinence by 11.0% (OR: 0.89, 95% CI 0.86–0.92, p < .0001) [45].

Participants’ perceptions of the addictiveness and harmfulness of e-cigarettes also influences intentions to quit. Those perceiving vaping to be less harmful or less addictive than smoking were significantly less likely to intend to quit. This emphasizes the need for more research evaluating the relative addictiveness and harms of vaping compared to smoking and developing education and health promotion strategies for current or future e-cigarette users.

The findings of this study will inform future expanded cross-sectional studies to better understand the diverse needs of current e-cigarette users. It will inform the need for research on understanding the relative harm and addictiveness of vaping compared to smoking. It identifies key factors that increase or decrease intention to quit, which clinicians and policymakers can understand and intervene upon. It also underscores the importance of developing robust, systematic, and diverse evidence-based interventions to support e-cigarette users who are interested to quit.

There are three important limitations of this study. First, this is a cross-sectional study with known and unknown confounders. While informative about the patterns and trends of current e-cigarette use, it cannot make definitive statements on causation. To improve the accuracy of our estimates, we conducted adjusted analyses to control for known confounders. Reassuringly, our findings also align with previous research in the area. Second, as this study recruited volunteers via online social media, there is potential for selection bias. Furthermore, since IP addresses were not collected, we cannot verify if all respondents were indeed from Ontario, so there is a possibility of geographical misrepresentation. Moreover, the short recruitment period and the use of a voluntary survey may have also limited the diversity and representativeness of the sample. This is a common issue among cross-sectional studies [46] and can affect generalization. Nonetheless, this improved feasibility by recruiting via mediums where there is a higher population of current e-cigarette users. It provides valuable information about the patterns of use of e-cigarette users, who are also typically younger and use social media [47]. E-cigarette manufactures have recognized this and have also attempted to target users via social media advertisement campaigns [48]. Future studies could aim to broaden the recruitment strategy to obtain a more representative sample. Third, it is important to recognize that the calculation for daily nicotine intake, which is used as a surrogate for plasma nicotine concentration, is crude and can vary based on the form of nicotine and how the e-cigarette is used [49]. Nonetheless, we concurrently measured nicotine dependence based on the modified Fagerstrom score, which has been validated among e-cigarette users, and correlated with daily nicotine intake [50]. The limitations in study design and methodology need to inform the conclusions drawn from this study. Nevertheless, these findings offer valuable insight into the patterns and perception of e-cigarette use, co-use of substances, and willingness to quit vaping among current e-cigarette users.

## 5 Conclusion

This study yields crucial insights into the patterns and perception of e-cigarette use, co-use of substances, and willingness to quit vaping among current e-cigarette users. It highlights high prevalence, nicotine consumption, and nicotine dependence, especially among youth and young adults, most of whom are using e-cigarettes recreationally rather than for smoking cessation. It is concerning that a large portion of respondents are using e-cigarettes to try and manage mental health issues while concurrently adding to addiction and mental health due to nicotine addiction. It is reassuring to see high interest to quit, especially among young individuals using nicotine in their e-cigarette with higher levels of nicotine dependence. However, lower perception of addictiveness and harm due to vaping appears to reduce intention to quit, highlighting an important opportunity for future research and education. There is a need to develop and evaluate tailored interventions to support individuals interested to quit vaping and it will be vital to take demographics, patterns of use, and perception of users into account for these vaping cessation interventions.

## Supporting information

S1 File Questionnaire, S2: Advertisements, S3 Table 1- S4 Table 2. (DOCX)
