# Supplementary material for: Patterns of e-cigarette use and interest in cessation among current users in Ontario: An online cross-sectional study
Source: PLoS One. 2025 May 9;20(5):e0322736. doi: 10.1371/journal.pone.0322736 (PMC12063819; doi:10.1371/journal.pone.0322736)
Supplement: S1 File — (DOCX) [file pone.0322736.s001.docx]

Supporting Information

S1: Questionnaire

A set of different advertising materials will be shown on the social media platforms, using 6 different images and three different types of phrasing (listed below).

Materials will be shown at the same frequency for a set period of time, so that their success (measured by conversion rate) can be compared. After this period of time, the most successful materials will be used for the remainder of the recruitment period, to optimize advertising costs. **Advertising images**

1) Vaping devices
2) Person vaping – neutral
3) Person vaping – young and producing vapor
4) Group of people vaping
5) Lung imagery (“concerning”)
6) Lung imagery (“neutral”)

**Phrases**

1) Do you vape?
2) Concerned about your vaping?
3) Do you vape? It could be harming your health

S2: Advertisements

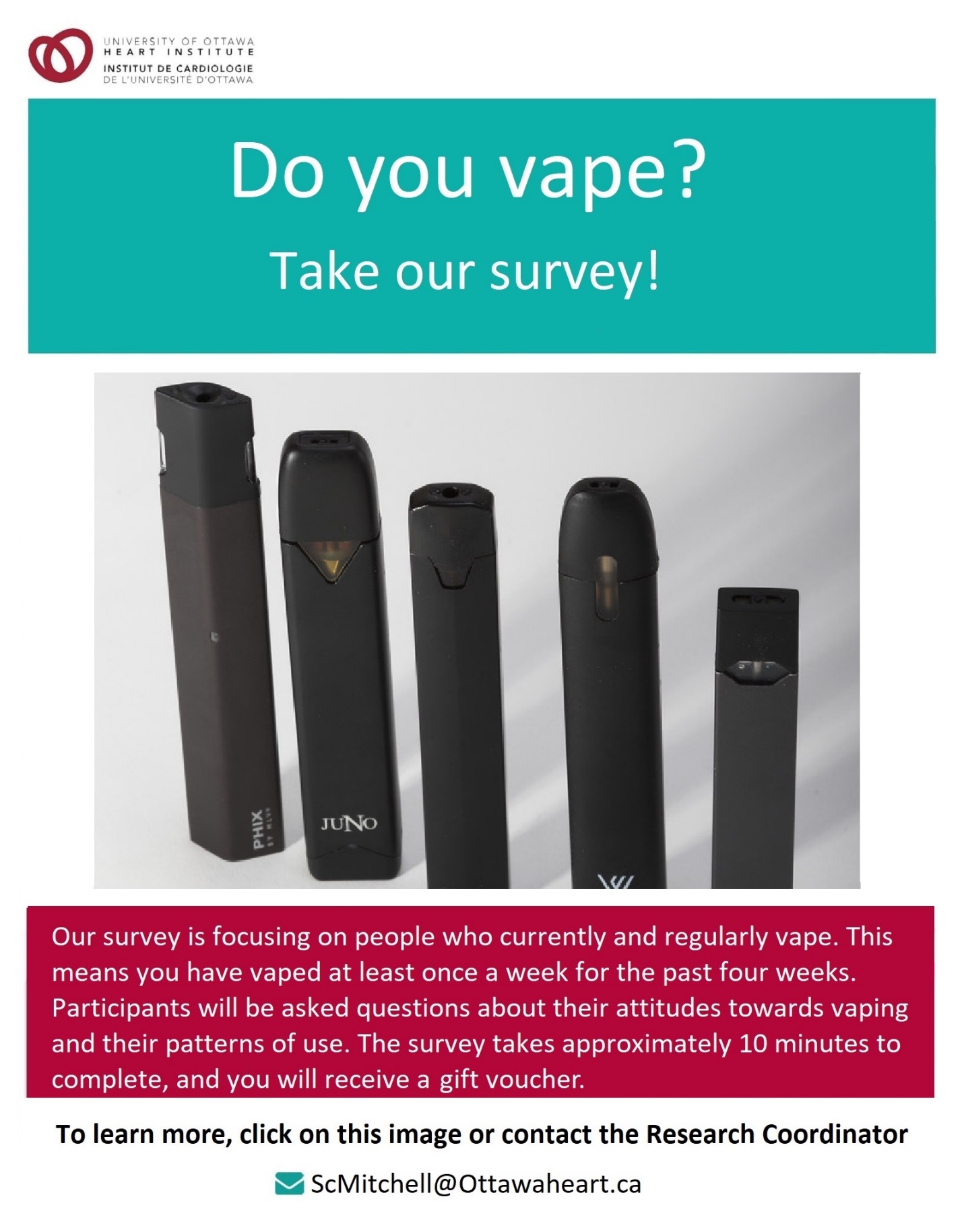


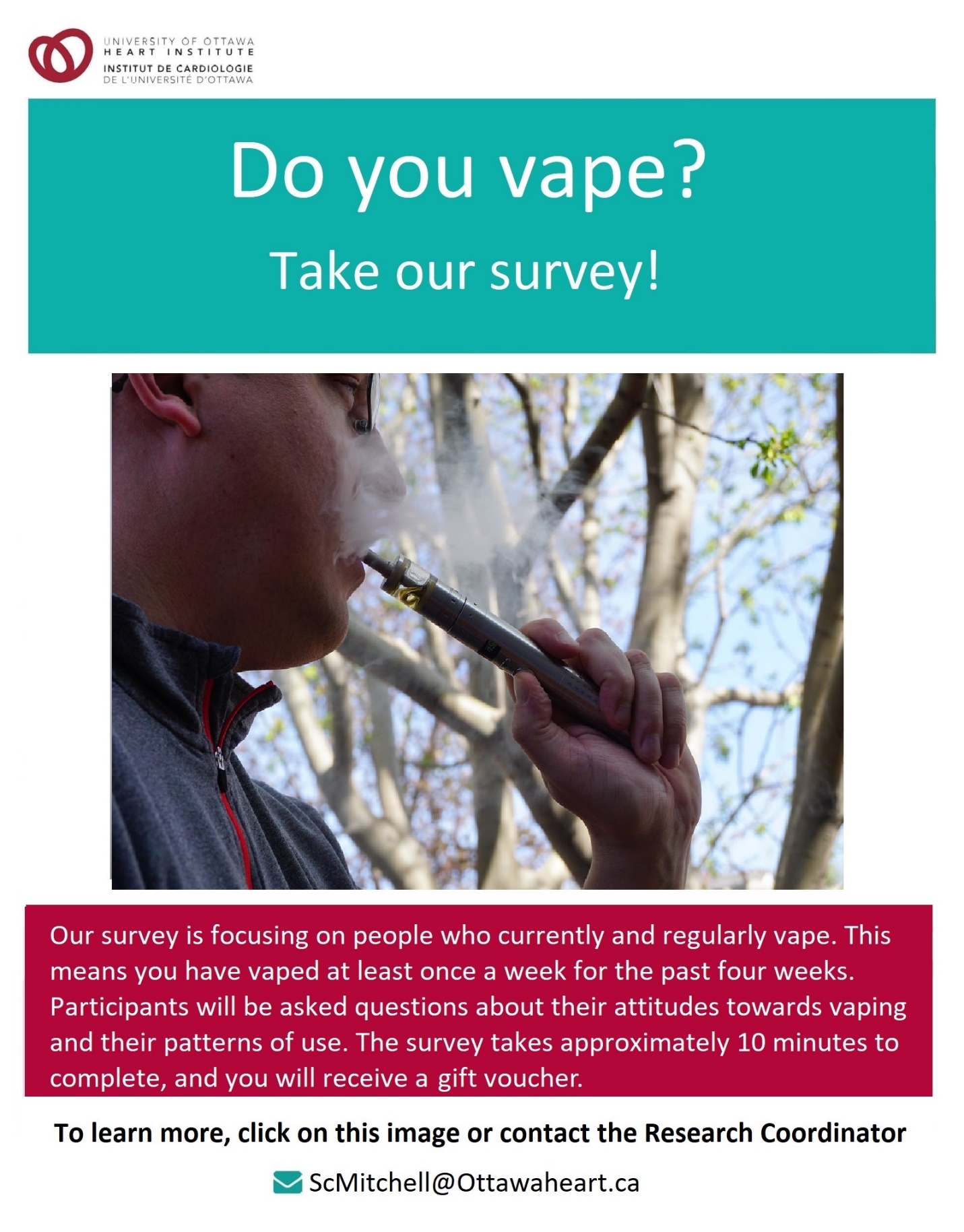


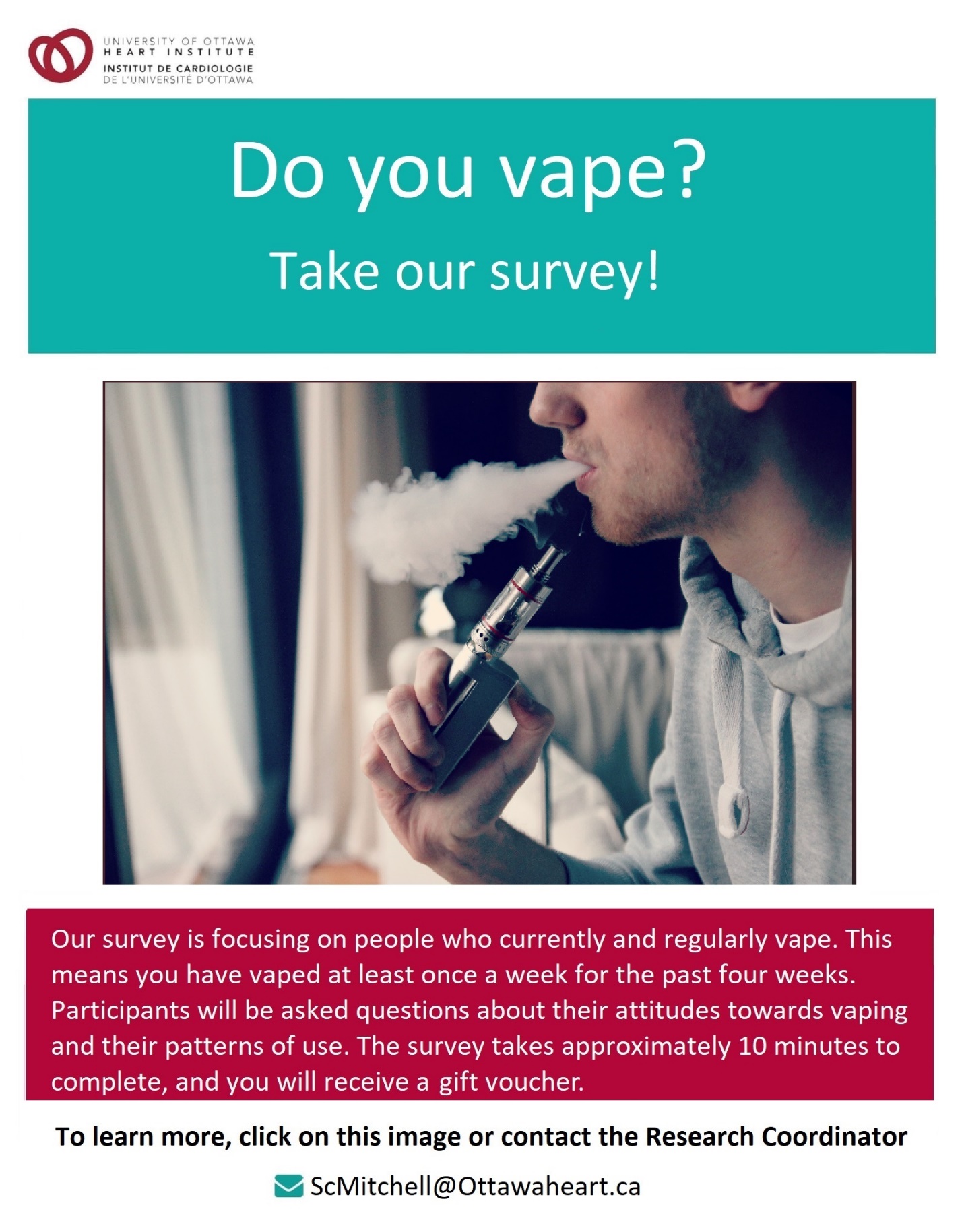


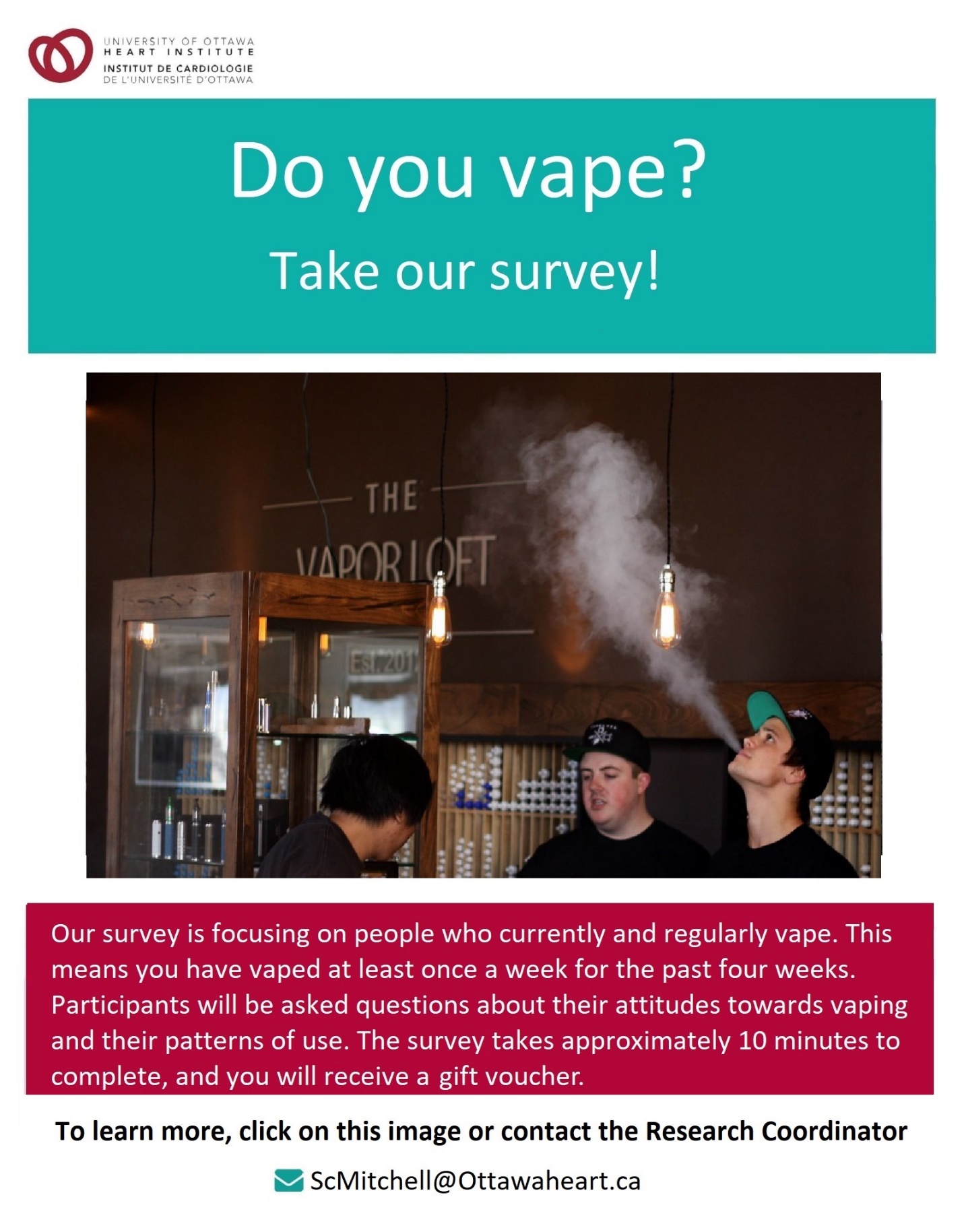


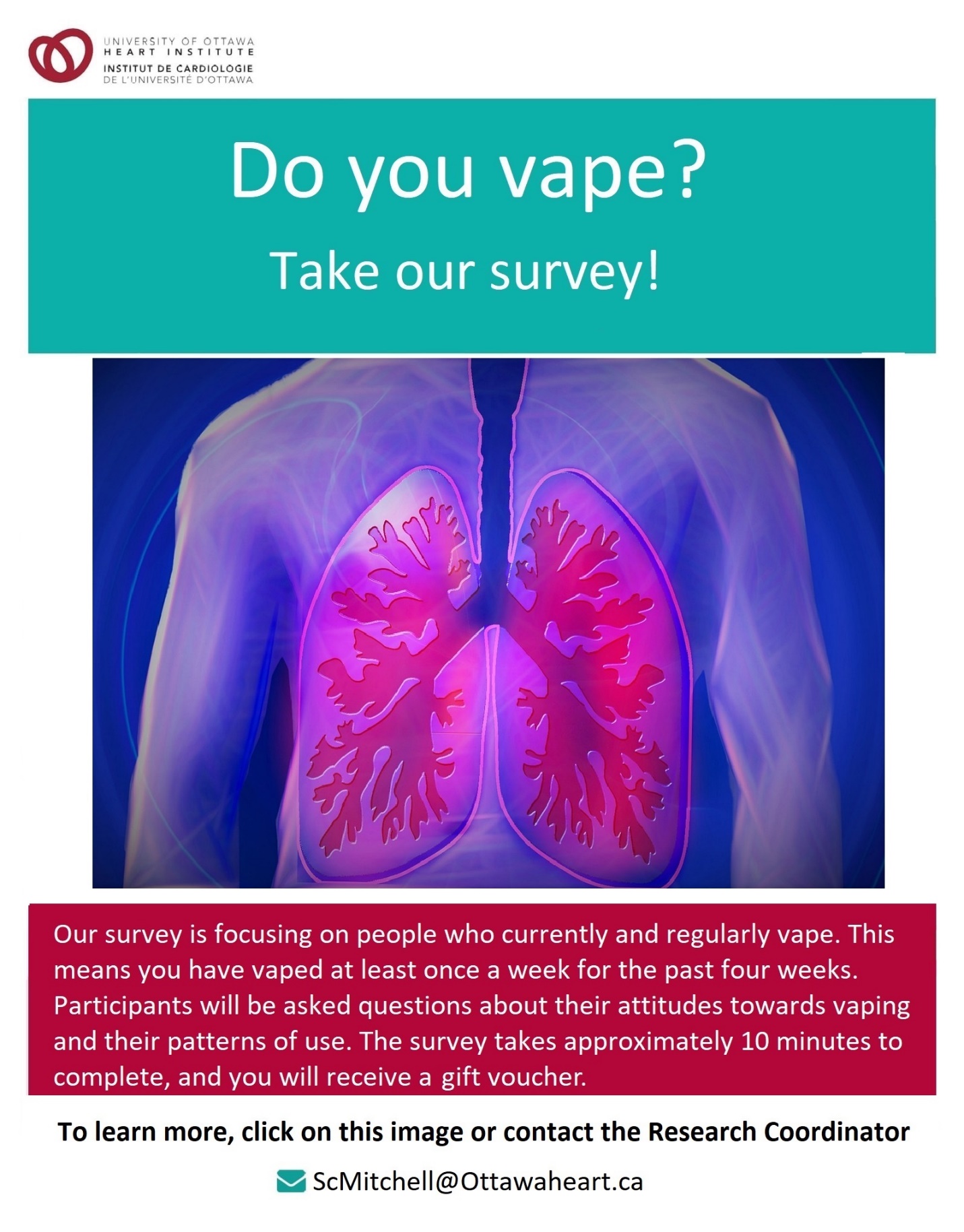


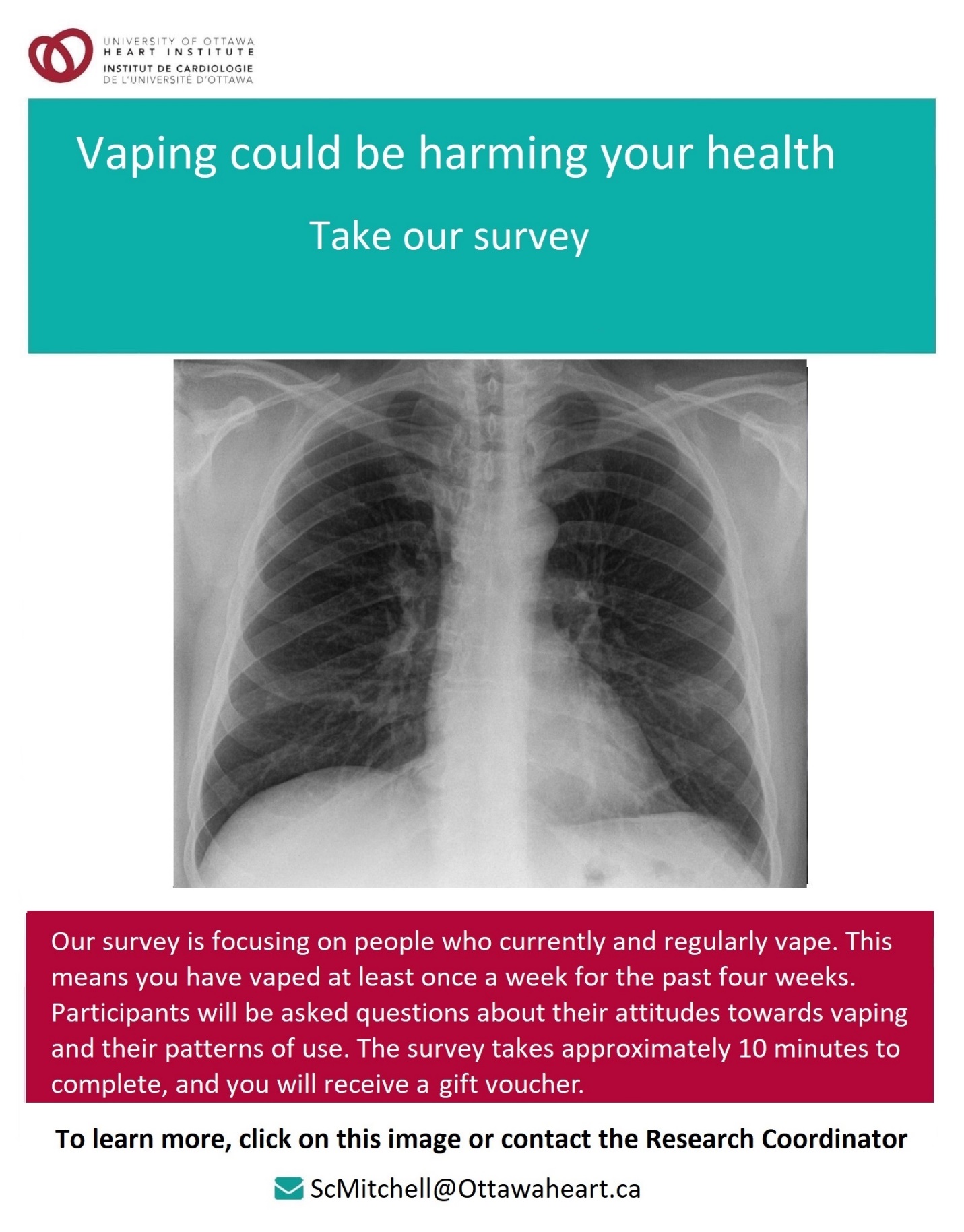


**S3 Table 1**. Patterns and characteristics of vaping and intention to quit by category.

|  | Total | Intention to quit (%)* | |
| --- | --- | --- | --- |
|  | N(%) | Yes | No |
| Age of first trying vaping (years old) |  |  |  |
| ≤ 18 | 220(29.1) | 95 (43.2) | 125(56.8) |
| 19 - 22 | 149(19.7) | 69(46.3) | 80(53.7) |
| 23 - 29 | 125(16.5) | 47(37.6) | 78(62.4) |
| 30 - 40 | 137(18.1) | 61(44.5) | 76(55.5) |
| 41 - 50 | 72(9.5) | 25(34.7) | 47(65.3) |
| 51 - 64 | 50(6.6) | 14(28.0) | 36(72) |
| 65+ | 4(0.5) | 2(50) | 2(50) |
|  |  |  |  |
| Age of vaping regularly (years old) |  |  |  |
| ≤ 19 | 262(34.6) | 121(46.2) | 141(53.8) |
| 20 - 24 | 146(19.3) | 67(45.9) | 79(54.1) |
| 25 - 34 | 156(20.6) | 54(34.6) | 102(65.4) |
| 35 - 44 | 96(12.7) | 42(43.8) | 54(56.3) |
| 45 - 54 | 55(7.3) | 19(34.5) | 36(65.5) |
| 55 - 64 | 37(4.9) | 9(24.3) | 28(75.7) |
| 65+ | 5(0.7) | 1(20.0) | 4(80.0) |
|  |  |  |  |
| Years of vaping regularly (years) |  |  |  |
| Less than 1 year | 124(16.4) | 46(37.1) | 78(62.9) |
| 1-2 years | 244(32.2) | 103(42.2) | 141(57.8) |
| 3-5 years | 294(38.8) | 144(49.0) | 150(51.0) |
| 6-9 years | 74(9.8) | 15(20.3) | 59(79.7) |
| 10+ years | 21(2.8) | 5(23.8) | 16(76.2) |
|  |  |  |  |
| Frequency of vaping per day |  |  |  |
| 0-9 times/day | 280(37) | 100(35.7) | 180(64.3) |
| 10-19 times/day | 239(31.6) | 117(49.0) | 122(51.0) |
| 20-29 times/day | 71(9.4) | 33(46.5) | 38(53.5) |
| 30+ times/day | 167(22.1) | 63(37.7) | 104(62.3) |
|  |  |  |  |
| Frequency of vaping overall |  |  |  |
| Every day | 616(81.4) | 256(41.6) | 360(58.4) |
| Every 2-3 days | 85(11.2) | 29(34.1) | 56(65.9) |
| Once a week | 35(4.6) | 15(42.9) | 20(57.1) |
| Twice a month | 17(2.2) | 10(58.8) | 7(41.2) |
| Once a month | 4(0.5) | 3(75.0) | 1(25.0) |
|  |  |  |  |
| Nicotine content of vaping device liquid |  |  |  |
| No nicotine | 80(10.6) | 16(20.0) | 64(80.0) |
| 3 - 15 mg/ml | 280(37) | 114(40.7) | 166(59.3) |
| 16 - 24 mg/ml | 246(32.5) | 123(50.0) | 123(50) |
| 25 - 49 mg/ml | 46(6.1) | 19(41.3) | 27(58.7) |
| ≥ 50 mg/ml | 76(10) | 31(40.8) | 45(59.2) |
| Do not know | 29(3.8) | 10(34.5) | 19(65.5) |
|  |  |  |  |
| Total amount of nicotine intake from vaping in a single day (mg nicotine) |  |  |  |
| No nicotine | 109(14.4) | 26(23.9) | 83(76.1) |
| 0.1-9.99 | 277(36.6) | 119(43.0) | 158(57) |
| 10-19.99 | 191(25.2) | 91(47.6) | 100(52.4) |
| 20-29.99 | 39(5.2) | 18(46.2) | 21(53.8) |
| 30-39.99 | 87(11.5) | 39(44.8) | 48(55.2) |
| ≥40 | 54(7.1) | 20(37.0) | 34(63) |
|  |  |  |  |
| Fagerstrom score (nicotine dependency) |  |  |  |
| Very low | 177(23.4) | 45(25.4) | 132(74.6) |
| Low | 195(25.8) | 82(42.1) | 113(57.9) |
| Moderate | 118(15.6) | 61(51.7) | 57(48.3) |
| High | 165(21.8) | 80(48.5) | 85(51.5) |
| Very high | 102(13.5) | 45(44.1) | 57(55.9) |

*Intention to quit percent is calculated by row

**S4 Table 2**: Logistic regression of intention to quit.

|  | | Crude | | Adjusted* | |
| --- | --- | --- | --- | --- | --- |
|  |  | OR (95 % CI) | P value | OR (95 % CI) | P value |
|  | **Age** |  |  |  |  |
|  | 15 - 17 |  | Reference |  |  |
|  | 18 - 24 | **2.021(1.176,3.474)** | **0.011** | **2.716(1.353,5.451)** | **0.005** |
|  | 25 - 34 | 1.48(0.835,2.624) | 0.179 | **3.903(1.453,10.489)** | **0.007** |
|  | 35 - 44 | **1.836(1.016,3.318)** | **0.044** | **5.783(1.638,20.408)** | **0.006** |
|  | 45 - 54 | 1.140(0.552,2.357) | 0.723 | 2.728(0.541,13.758) | 0.224 |
|  | 55 - 64 | 0.805(0.362,1.790) | 0.594 | 0.506(0.053,4.797) | 0.552 |
|  | 65+ | 0.181(0.022,1.469) | 0.110 | 0.086(0.003,2.250) | 0.141 |
|  |  |  |  |  |  |
|  | **Gender** |  |  |  |  |
|  | Male |  | Reference |  |  |
|  | Female | 0.824(0.611,1.112) | 0.206 | 1.013(0.71,1.445) | 0.943 |
|  |  |  |  |  |  |
|  | **Age of first try vaping** |  |  |  |  |
|  | ≤ 18 |  | Reference |  |  |
|  | 18 - 22 | 1.135(0.747,1.724) | 0.553 | 0.697(0.354,1.374) | 0.297 |
|  | 23 - 29 | 0.793(0.506,1.243) | 0.312 | 0.614(0.219,1.725) | 0.355 |
|  | 30 - 40 | 1.056(0.687,1.623) | 0.803 | 0.925(0.282,3.031) | 0.898 |
|  | 41 - 50 | 0.700(0.402,1.218) | 0.207 | 0.835(0.184,3.790) | 0.816 |
|  | 51 - 64 | 0.512(0.261,1.002) | 0.051 | 4.347(0.497,37.991) | 0.184 |
|  | 65+ | 1.316(0.182,9.511) | 0.786 | 6.276(0.202,194.88) | 0.295 |
|  |  |  |  |  |  |
|  |  |  |  |  |  |
|  | **Age of vaping regularly** |  |  |  |  |
|  | ≤19 |  | Reference |  |  |
|  | 20 - 24 | 0.988(0.658,1.483) | 0.955 | 1.184(0.594,2.363) | 0.631 |
|  | 25 - 34 | **0.617(0.410,0.929)** | **0.021** | 0.735(0.276,1.956) | 0.537 |
|  | 35 - 44 | 0.906(0.566,1.451) | 0.682 | 0.690(0.215,2.217) | 0.533 |
|  | 45 - 54 | 0.615(0.335,1.128) | 0.116 | 1.518(0.328,7.038) | 0.594 |
|  | 55 - 64 | **0.375(0.170,0.825)** | **0.015** | 1.183(0.169,8.299) | 0.866 |
|  | 65+ | 0.291(0.032,2.642) | 0.273 | 1.470(0.062,34.912) | 0.812 |
|  |  |  |  |  |  |
|  | **Nicotine concentration of e-liquid** |  |  |  |  |
|  |  |  |  |  |  |
|  | No nicotine |  | Reference |  |  |
|  | 3 - 15 mg/ml | **2.747(1.511,4.993)** | **0.001** | **2.691(1.295,5.593)** | **0.008** |
|  | 16 - 24 mg/ml | **4.000(2.191,7.304)** | **<0.001** | **3.096(1.48,6.479)** | **0.003** |
|  | 25 - 49 mg/ml | **2.815(1.261,6.282)** | **0.012** | 2.426(0.937,6.283) | 0.068 |
|  | 50 mg/ml or more | **2.756(1.350,5.626)** | **0.005** | 2.171(0.881,5.348) | 0.092 |
|  | I do not know | 2.105(0.821,5.398) | 0.121 | 2.462(0.832,7.282) | 0.104 |
|  |  |  |  |  |  |
|  |  |  |  |  |  |
|  | **Frequency of vaping** |  |  |  |  |
|  | 0-9 times/day |  | Reference |  |  |
|  | 10-19 times/day | **1.726(1.214,2.455)** | **0.002** | 1.369(0.863,2.172) | 0.182 |
|  | 20-29 times/day | 1.563(0.923,2.647) | 0.096 | 0.905(0.445,1.839) | 0.782 |
|  | 30+ times/day | 1.090(0.733,1.622) | 0.669 | 0.629(0.326,1.214) | 0.167 |
|  |  |  |  |  |  |
|  |  |  |  |  |  |
|  | **Perception of harm to health of vaping compared to smoking cigarettes** |  |  |  |  |
|  | More harmful to health |  | Reference |  |  |
|  | About the same in terms of health impact | **0.495(0.250,0.979)** | **0.043** | 0.690(0.311,1.531) | 0.362 |
|  | Less harmful to health | **0.242(0.128,0.457)** | **<0.001** | **0.372(0.170,0.815)** | **0.013** |
|  | Not sure | **0.355(0.159,0.793)** | **0.012** | 0.613(0.236,1.591) | 0.314 |
|  |  |  |  |  |  |
|  |  |  |  |  |  |
|  | **Perception of addictiveness of vaping compared to smoking cigarettes** |  |  |  |  |
|  | More addictive |  | Reference |  |  |
|  | About the same level of addictiveness | **0.383(0.265,0.553)** | **<0.001** | **0.373(0.241,0.576)** | **<0.001** |
|  | Less addictive | **0.253(0.170,0.377)** | **<0.001** | **0.258(0.156,0.427)** | **<0.001** |
|  | Not sure | **0.333(0.135,0.821)** | **0.017** | 0.359(0.127,1.012) | 0.053 |
|  |  |  |  |  |  |
|  |  |  |  |  |  |
|  | **Frequency of tobacco use** |  |  |  |  |
|  | Do not smoke |  | Reference |  |  |
|  | Every day | **2.012(1.267,3.195)** | **0.003** | 1.303(0.753,2.256) | 0.345 |
|  | Every 2-3 days | 1.753(0.918,3.346) | 0.089 | 1.014(0.474,2.173) | 0.971 |
|  | Once a week | **2.235(1.301,3.841)** | **0.004** | 1.419(0.766,2.629) | 0.265 |
|  | Once a month | 1.568(0.866,2.838) | 0.137 | 0.780(0.389,1.564) | 0.483 |
|  | Less than once a month | 1.322(0.873,2.003) | 0.187 | 0.965(0.601,1.548) | 0.883 |
|  |  |  |  |  |  |
|  | **Frequency of cannabis use** |  |  |  |  |
|  | Do not consume cannabis |  | Reference |  |  |
|  | Every day | 1.140(0.797,1.632) | 0.472 | 0.978(0.645,1.481) | 0.915 |
|  | Every 2 - 3 days | 1.545(0.976,2.447) | 0.063 | 1.324(0.756,2.318) | 0.326 |
|  | Once a week | 1.014(0.498,2.068) | 0.968 | 0.862(0.376,1.976) | 0.726 |
|  | 1 - 3 times a month | 1.232(0.626,2.426) | 0.546 | 1.281(0.582,2.818) | 0.538 |
|  | Less than once a month | 1.574(0.922,2.687) | 0.096 | 1.656(0.898,3.052) | 0.106 |
|  |  |  |  |  |  |
|  |  |  |  |  |  |
|  | **Fagerstrom Score (Categorized)** |  |  |  |  |
|  | Very low |  | Reference |  |  |
|  | Low | **2.226(1.440,3.439)** | **<0.001** | **1.869(1.105,3.163)** | **0.020** |
|  | Moderate | **3.001(1.830,4.923)** | **<0.001** | **2.474(1.306,4.689)** | **0.005** |
|  | High | **2.725(1.741,4.267)** | **<0.001** | **2.268(1.206,4.267)** | **0.011** |
|  | Very high | **2.286(1.372,3.808)** | **0.001** | 2.025(0.904,4.536) | 0.086 |

* Adjusted for age, sex, first-time vaping ever, first time of regular vaping, nicotine content of the vaping device, tobacco smoking, cannabis consumption, and Fagerstrom score.
